# Prediction of initial objective response to drug-eluting beads transcatheter arterial chemoembolization for hepatocellular carcinoma using CT radiomics-based machine learning model

**DOI:** 10.3389/fphar.2024.1315732

**Published:** 2024-01-25

**Authors:** Xueying Zhang, Zijun He, Yucong Zhang, Jian Kong

**Affiliations:** ^1^ The Second Clinical Medical College, Jinan University, Shenzhen, Guangdong, China; ^2^ Department of Radiation Oncology, Shenzhen People’s Hospital (Second Clinical Medical College of Jinan University, First Affiliated Hospital of Southern University of Science and Technology), Shenzhen, Guangdong, China; ^3^ Department of Interventional Radiology, Shenzhen People’s Hospital (Second Clinical Medical College of Jinan University, First Affiliated Hospital of Southern University of Science and Technology), Shenzhen, Guangdong, China

**Keywords:** hepatocellular carcinoma, drug-eluting beads transcatheter arterial chemoembolization, initial response, objective response, computed tomography, radiomics, nomogram, prediction

## Abstract

**Objective:** A prognostic model utilizing CT radiomics, radiological, and clinical features was developed and validated in this study to predict an objective response to initial transcatheter arterial chemoembolization with drug-eluting beads (DEB-TACE) for hepatocellular carcinoma (HCC).

**Methods:** Between January 2017 and December 2022, the baseline clinical characteristics and preoperative and postoperative follow-up imaging data of 108 HCC patients who underwent the first time treatment of DEB-TACE were analyzed retrospectively. The training group (*n* = 86) and the validation group (*n* = 22) were randomly assigned in an 8:2 ratio. By logistic regression in machine learning, radiomics, and clinical-radiological models were constructed separately. Finally, the integrated model construction involved the integration of both radiomics and clinical-radiological signatures. The study compared the integrated model with radiomics and clinical-radiological models using calibration curves, receiver operating characteristic (ROC) curves, and decision curve analysis (DCA).

**Results:** The objective response rate observed in a group of 108 HCC patients who received initial DEB-TACE treatment was found to be 51.9%. Among the three models, the integrated model exhibited superior predictive accuracy in both the training and validation groups. The training group resulted in an area under the curve (AUC) of 0.860, along with sensitivity and specificity values of 0.650 and 0.913, respectively. Based on the findings from the validation group, the AUC was estimated to be 0.927. Additionally, it was found that values of sensitivity and specificity were 0.875 and 0.833, respectively. In the validation group, the AUC of the integrated model showed a significant improvement when contrasted to the clinical-radiological model (*p* = 0.042). Nevertheless, no significant distinction was observed in the AUC when comparing the integrated model with the radiomics model (*p* = 0.734). The DCA suggested that the integrated model demonstrates advantageous clinical utility.

**Conclusion:** The integrated model, which combines the CT radiomics signature and the clinical-radiological signature, exhibited higher predictive efficacy than either the radiomics or clinical-radiological models alone. This suggests that during the prediction of the objective responsiveness of HCC patients to the first DEB-TACE treatment, the integrated model yields superior outcomes.

## 1 Introduction

Primary liver cancer is globally recognized as it holds the third position in terms of frequency as a leading cause of cancer-related mortality and is acknowledged as the sixth most common cancer overall, with hepatocellular carcinoma (HCC) accounting for about 75%–85% of them (Sung et al., 2021). Transarterial chemoembolization (TACE) is endorsed as the standard treatment for intermediate-stage HCC by the European Society for Medical Oncology (ESMO), the American Association for the Study of Liver Diseases (AASLD), the Barcelona Clinic Liver Cancer (BCLC), and various other guidelines (Heimbach et al., 2018; Vogel et al., 2021; Reig et al., 2022). Additionally, TACE can be utilized for patients with early and advanced HCC (Han and Kim, 2015). Depending on the embolic agent, TACE can be classified into the following (Kotsifa et al., 2022): (i) conventional TACE (cTACE), which uses lipiodol and gelatin sponge particles or polyvinyl alcohol particles; and (ii) drug-eluting beads transcatheter arterial chemoembolization (DEB-TACE), which uses drug-eluting microspheres. DEB-TACE is widespread in its clinical applications because it offers a higher level of safety and standardization of procedure compared to cTACE.

Because of the high heterogeneity of the patient population, the efficacy and safety of TACE treatment for patients experiencing intermediate-stage HCC may vary (Kudo et al., 2020). Therefore, studying the subgroups of intermediate-stage HCC patients is a challenging and popular research topic. Although the 2022 BCLC guidelines further subdivide BCLC stage B (Reig et al., 2022), the boundaries between the three subgroups are blurred, and the scope of application of TACE is vague. Thus, it is important to have an objective way of predicting response to TACE treatments in patients with HCC before treatment begins.

In recent years, using radiomics for the prediction of effectiveness and prognosis of TACE for HCC has garnered increasing interest (Kim et al., 2018; Kong et al., 2021; Zhao et al., 2021; Wang et al., 2022; Sun et al., 2023). Radiomics is a non-invasive imaging method that can assess tumor size, shape, texture, and other characteristics, providing quantitative, high-dimensional, and mineable features for further analysis (Varghese et al., 2019; Chen et al., 2021). In addition, radiomic features, as a combination of multiple features, are considered a more powerful prognostic biomarker, providing additional information for clinical data and reported to be an important predictive factor for clinical outcomes (Coroller et al., 2015; Huang et al., 2016). Additionally, machine learning methods can accurately handle complex relationships between a large number of variables, which is difficult to achieve with traditional statistical models (Choy et al., 2018). However, there is a paucity of studies on the radiomics of DEB-TACE for HCC. Therefore, our main aim is to create and validate predictive models, specifically a radiomics model, clinical-radiological model, and integrated model. These models depended on clinical, radiological, and CT radiomics characteristics. The purpose of these models was to mediate the preoperative identification of patients with HCC who would derive the greatest advantage from initial DEB-TACE and to anticipate patients prognoses.

## 2 Material and methods

### 2.1 Research ethics and study participants

The requirement for informed consent was waived due to the retrospective nature of the investigation. Shenzhen People’s Hospital’s Institutional Review Board approved the study (IRB No. LL-KY-2022137-01, Shenzhen, China) and registered it with the Chinese Clinical Trial Register (ChiCTR2200060448, China). Helsinki Declaration principles were followed in the conduct of the investigation.

**FIGURE 1:**
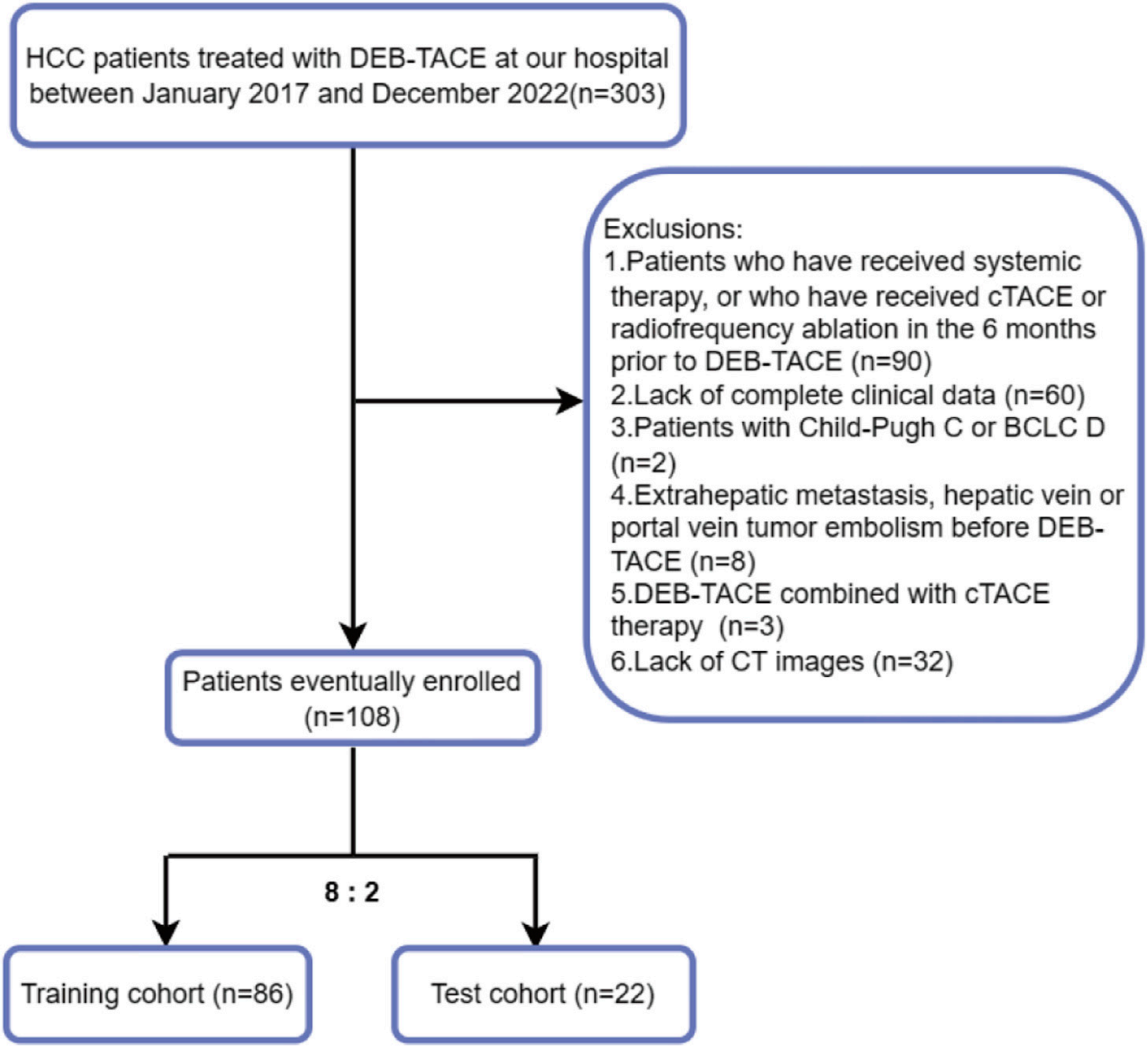
Flowchart of inclusion and exclusion criteria.

The criteria for inclusion were as follows: (i) patients with definitive clinical or histological diagnosis of HCC in accordance with the 2022 guidelines established by the Chinese Society of Clinical Oncology (CSCO) for the identification and management of primary liver cancer (Zhou et al., 2023); (ii) patients aged 18–85 years; (iii) patients with BCLC stage B without surgical indications or those with BCLC stage A unable to undergo/refused curative therapies (surgical resection, liver transplantation, or radiofrequency ablation); (iv) those with Child–Pugh liver function score of A5-B7; (v) patients with a score of 0 on the Eastern Cooperative Oncology Group (ECOG); and (vi) those that underwent DEB-TACE as primary treatment or without cTACE/ablation within 6 months before initial DEB-TACE. The criteria for exclusion were as follows: (i) patients with HCC having spontaneous rupture and hemorrhage; (ii) those for whom current treatment was integrated with any other systemic or local treatment for HCC; (iii) those with lack of baseline clinical data or liver CT scan and enhancement imaging data; (iv) those with infiltrative/diffuse HCC (Reig et al., 2022); (v) those that had lesions without arterial phase enhancement, where the largest lesions were smaller than 1 cm; (vi) those with uncontrolled organ dysfunction or metabolic disease; (vii) those with incomplete data over the follow-up period; and (viii) patients that had images of poor quality due to scanning artifacts. Follow-up imaging data at 4–6 weeks after the initial DEB-TACE procedure was used as the study endpoint. Retrospective collection of clinical and imaging data was conducted on HCC patients who received admission to our interventional department for their initial treatment with DEB-TACE between the period of January 2017 and December 2022. Ultimately, in the study, a total of 108 patients were included and were randomly allocated to either the training group (*n* = 86) or the validation group (*n* = 22) at an 8:2 ratio. Figure 1 depicts a flowchart illustrating the inclusion and exclusion criteria.

### 2.2 DEB-TACE procedure

TACE procedures were conducted by two physicians with over 10 years of independent experience and special training in interventional radiology. Under local anesthesia, the Seldinger technique was used for femoral artery cannulation. Angiography of the abdominal aorta was performed first, followed by angiography of the celiac artery, superior mesenteric artery, and common hepatic artery to observe whether the tumor had any vascular variants or parasitic blood supply and whether the main trunk of the portal vein and its branches were patent. Using an angiogram, superselective cannulation of the tumor’s supplying artery was carried out, allowing the identification of target vessels. Appropriate particle size (70–150 μm/100–300 μm/300–500 μm) of CalliSpheres^®^ beads (Hengrui Medical, Suzhou, China) or DC^®^ beads (Biocompatibles UK Ltd., Farnham, United Kingdom) and dose of embolization (1-2 vial) were selected depending on tumor size, liver function score, and degree of vascular enhancement (Shao et al., 2021). Following the manufacturer’s guidelines (Lencioni et al., 2012), the microspheres were loaded with chemotherapeutic drugs (pirarubicin, 50–75 mg per 2 mL/vial beads). Subsequently, they were mixed with a non-ionic contrast agent, iophorol-350 (Hengrui Medical, Suzhou, China), in a volume of 10–15 mL. The mixture was then slowly injected under fluoroscopy at a rate of 1 mL/min (Lencioni et al., 2012). A final postoperative angiogram was performed to determine the embolization endpoint, which was assessed based on the subjective angiographic chemoembolization endpoint (SACE). Ideal endpoints for embolization include the absence of tumor blush, the reduction of antegrade arterial flow, and the appearance of “dead branches” of the main tumor vessel (i.e., SACE stage III). Postoperative symptomatic treatment such as hepatoprotective agents, analgesia, and antiemetics were routinely administered.

### 2.3 CT scanning

In all patients in the study, dynamic liver CT was performed before and 4–6 weeks after DEB-TACE, including plain, arterial, portal vein, and delayed-phase imaging. The CT and enhanced CT scanning of the liver was performed using the SOMATOM Definition AS 16-row spiral CT (SIEMENS Healthineers, Erlangen, Germany), Philips Brilliance 16-row spiral CT (manufactured by Philips Healthcare, Cleveland, OH, United States), or Philips Brilliance iCT 256-slice spiral CT (manufactured by Philips Healthcare, Cleveland, OH, United States) devices. This study employed the subsequent scanning parameters: Pitch value ranged from 1.3 to 1.5, slice thickness was set at 5 mm, tube voltage was set at 120 kV, automatic tube current modulation was used, and reconstruction interval was set at 1.5 mm.

### 2.4 Clinical data, radiological features, and DSA image feature definitions

Baseline demographic, laboratory, imaging, and intraoperative DSA imaging feature data were retrospectively collected. These included age, gender, etiology, BCLC stage, Child–Pugh grade, albumin-bilirubin (ALBI) grade, serum albumin, serum total bilirubin, platelet count, alpha-fetoprotein, PT extension time score, lobar involvement, microsphere size, presence of vascular lakes, presence of capsule, vascularization patterns, SACE grade, maximum tumor diameter, number of tumors, and six-and-twelve score. Patient imaging data was collected 4–6 weeks following initial treatment.

The examination of the tumor’s response to treatment in relation to the target lesions was carried out utilizing the mRECIST criteria (Lencioni and Llovet, 2010). The assessment categorized the response into one of four classifications: complete response (CR), partial response (PR), stable disease (SD), or progressive disease (PD). Objective response (OR) includes CR and PR. The objective response rate (ORR) was calculated as CR rate + PR rate.

Baseline CT radiological features were defined as follows. (i) Six-and-twelve score, with the tumor burden value represented as the “maximum diameter of the largest lesion (cm) + the number of lesions” (Wang et al., 2019), categorized into three classes: ≤6, >6 but ≤12, and >12. (ii) Lobar involvement was described using the Couinaud classification of liver anatomy. A tumor confined to segments S5-8 (right lobe), segments S2 and S3 (left lobe), or segments S1 and S4 (caudate and quadrate lobes) was defined as a unilobar tumor; all other tumors were defined as bilobar tumors (Vesselle et al., 2016). (iii) Vascularization patterns were classified into four types based on dynamic enhanced CT before treatment (Kawamura et al., 2010). There are four types of enhancement patterns observed in this study. Type 1 exhibits a homogeneous pattern of enhancement without any elevation in arterial blood flow. Type 2 also shows a homogeneous pattern of enhancement but with an elevation in arterial blood flow. Type 3 displays a heterogeneous pattern of enhancement with the presence of septations. Lastly, type 4 exhibits a heterogeneous pattern of enhancement characterized by irregular ring-like structures. The present study grouped these types into two categories: types 1 + 2 and 3 + 4 (Hu et al., 2020).

Laboratory test results were defined as follows: (i) ALBI (Zheng et al., 2017) was determined as $\log_{10}\text{bilirubin }\left(\mu \text{mol}/L\right)\times 0.66+\text{albumin }\left(g/L\right)\times -0.085$ , scored as “1” for values of ≤ −2.60, “2” for > −2.60 and ≤ −1.39, and “3” for > −1.39. (ii) Prothrombin (PT) extension time score (Durand and Valla, 2008) was calculated as measured PT − control PT, and it was scored as “1” for 1–3 s, “2” for 4–6 s, and “3” for >6 s.

The following was the definition of perioperative angiography. (i) The endpoint of embolization was classified into four grades based on the SACE (Lewandowski et al., 2007): In SACE I, there is a presence of normal arterial blood flow and a decrease in tumor blush. In SACE II, there is a decrease in both arterial blood flow and tumor blush. The patient’s condition is characterized by SACE III, which is associated with diminished arterial blood flow and the absence of a tumor blush. The SACE IV classification indicates the absence of arterial blood flow or tumor blush. (ii) Vascular lakes (Kong et al., 2020) were defined as localized accumulations of contrast agents in the tumor during embolization persisting to the venous phase without dissipation, similar to extravasation but different from tumor blush.

The initial response to tumor therapy and the radiological characteristics mentioned above were assessed by two diagnostic radiologists who were blinded to the clinical information. All instances of disagreement were effectively resolved through the process of reaching a consensus. The reliability of the data was assessed using Cohen’s Kappa test.

### 2.5 Image segmentation

The manual segmentation of the arterial phase in the liver involved the segmentation of each layer of the target lesions was conducted using the ITK-SNAP software (version 3.8.0, http://www.itksnap.org) (Park et al., 2017). All liver tumor images were segmented by a radiologist with over 5 years of professional experience in liver CT diagnosis, who performed the task independently and without access to any clinical information about the patient. In cases where the tumor margins were blurred, the outline of the tumor was determined in our hospital’s routine clinical records and picture archiving and communication systems (PACS) by observing the arterial, portal, and delayed phase images. The verification of the volume of interest (VOIs) was subsequently conducted by an additional radiologist possessing a decade of diagnostic expertise in liver CT imaging. In order to measure the reproducibility of radiomics characteristics, the application of intra-observer reproducibility analysis was utilized. A radiologist randomly performed image segmentation of 50 cases at two-time points at 1-month intervals, generating 2 VOIs for each included patient. Intraclass correlation coefficients (ICCs) were used to assess the agreement between extracted features. Radiomics features with ICCs of ≥0.75 were considered to have good reproducibility and stability. The radiomics features extracted in this study had good reproducibility and stability, with an intra-observer ICC between 0.93 and 0.99 based on two measurements.

### 2.6 Feature extraction, feature selection, and radiomics model construction

**FIGURE 2:**
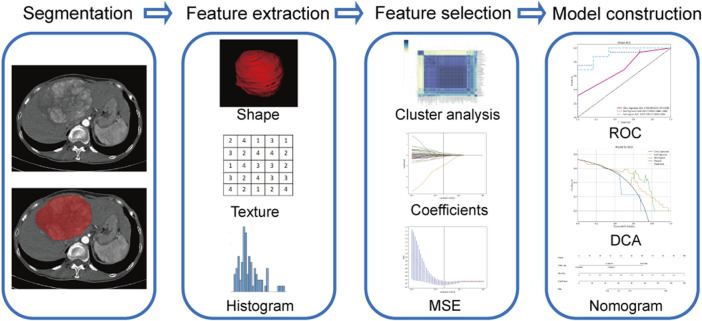
Flowchart of radiomics model construction. MSE, means square error; ROC, receiver operating characteristic; DCA, decision curve analysis.

Using Pyradiomic’s in-house feature analysis program (http://pyradiomics.readthedocs.io), all radiomics features were extracted. First, the Z-score method was employed to standardize all features, involving the calculation of the mean and variance for each feature column. Subsequently, each feature column was transformed into a standard normal distribution by subtracting the mean and dividing by the variance. Next, the statistical tests employed for the purpose of identifying features exhibiting significant differences were the *t*-test and the Mann-Whitney U-test (*p* < 0.05), and we retained 355 features with *p*-values less than 0.05. Then, Spearman’s rank correlation coefficient was employed to ascertain the correlation between the features for redundancy elimination. When the correlation coefficient between any two features was ≥0.9, only one of the two features was retained. We adopt a greedy recursive deletion approach to filter features, wherein we remove the features with the highest redundancy in the current set at each iteration, resulting in the retention of 56 final features. Finally, the most robust and non-redundant features were filtered by the least absolute shrinkage and selection operator (LASSO) regression with 10-fold cross-validation. All feature screening processes are performed in the training group. The retained features were then utilized in machine learning for risk modeling by LR in scikit-learn machine learning library. To prevent overfitting, a 5-fold cross-validation was employed to select the optimal parameters of the model, and to obtain final radiomics signatures. Figure 2 shows a flowchart of the radiomics analysis in the present study, including lesion segmentation, feature extraction, feature selection, and model construction.

### 2.7 Clinical-radiological and integrated model construction

The clinical and radiological features were assessed using statistical tests such as the *t*-test, Mann-Whitney U-test, Chi-square test, or Fisher’s exact test. The features that had a significance level of <0.05 were encompassed in the logistic regression model for the purpose of constructing the clinical-radiological model in the field of machine learning, similar to the aforementioned approach. Ultimately, the integration of radiomics and clinical-radiological signatures was employed to establish an integrated model.

### 2.8 Model performance, validation, and comparison

The evaluation of model discrimination was conducted through the utilization of ROC analysis. The calculation of the area under the curve (AUC) of the ROC was performed, and Delong’s test was employed to compare the AUC values between the models. The evaluation of the prediction model’s calibration was conducted by employing the Hosmer-Lemeshow test and presenting the results through calibration curves. A *p* > 0.05 indicates a favorable level of concordance between the anticipated values and the observed values of the model. The assessment of the model’s clinical utility was conducted by DCA.

### 2.9 Logistic regression

Logistic regression is a statistical model commonly used to test hypotheses about the relationships between a dichotomous response and one or more categorical or continuous explanatory variables. The fitted line plot in logistic regression has a sigmoid or S-shaped curve, which is more appropriate for representing the data compared to the linear regression line. Logistic regression uses the logit function, which is the natural logarithm, to model the relationship between variables (Demirsoy and Karaibrahimoglu, 2023). For more information on logistic regression, refer to the study by Peng et al. (2002).

### 2.10 Statistics

The statistical methods employed for analyzing the disparities in the clinical and radiological characteristics of participants were the *t*-test, or the Mann-Whitney U test, and the Chi-square test, or Fisher’s exact test. Quantitative variables are expressed as‾x ± s; categorical variables are expressed using frequencies (percentages). IBM SPSS Statistics (Version 21.0) was used to analyze clinical and radiological features statistically. Python (version 3.7.16) was used for statistical analysis of radiomic features, ICCs, Z-score normalization, *t*-test or Mann–Whitney *U*-test, Spearman rank correlation test, LASSO regression, and machine learning. Statistical significance was determined for variations with a *p*-value less than 0.05. Subjective radiological features were tested for consistency using Cohen’s Kappa test. The Cohen’s Kappa values were classified as follows: poor, <0.20; general, 0.21–0.40; moderate, 0.41–0.60; good, 0.61–0.80; very good, >0.81.

## 3 Results

### 3.1 Basic characteristics

Table 1 displays the baseline characteristics of the patients in both the training and validation groups.

**TABLE 1:** The baseline characteristics of the patients in the training and validation groups.

	Training Cohort(*n* = 86)			Test cohort (*n* = 22)		
Characteristics	Without OR	OR	*p*-value	Without OR	OR	*p*-value
**Age (years)**	58.28 ± 12.84	58.40 ± 15.32	0.969	66.67 ± 7.42	55.88 ± 14.45	0.100
**Serum albumin (g/L)**	38.57 ± 6.14	38.81 ± 5.20	0.851	33.03 ± 5.83	39.37 ± 3.93	0.008
**Serum total bilirubin (μmol/L)**	20.33 ± 18.56	17.45 ± 8.92	0.373	21.93 ± 8.44	16.06 ± 6.59	0.099
**PLT (×10** ^**9**^ ** ** **g/L)**	200.88 ± 124.29	156.25 ± 82.50	0.057	247.17 ± 141.07	164.50 ± 57.88	0.060
**Maximum tumour diameter (cm)**	6.82 ± 5.04	5.72 ± 3.95	0.266	10.42 ± 5.59	4.04 ± 2.79	0.002
**Tumor number**	3.00 ± 2.67	2.35 ± 2.80	0.274	3.67 ± 4.08	1.88 ± 1.26	0.121
**Gender**			0.567			0.876
Female	4(8.70)	6(15.00)		2(33.33)	3(18.75)	
Male	42(91.30)	34(85.00)		4(66.67)	13(81.25)	
**Etiology**			0.099			0.805
Others	13(28.26)	4(10.00)		1(16.67)	2(12.50)	
HBV	31(67.39)	33(82.50)		5(83.33)	13(81.25)	
HCV	2(4.35)	3(7.50)		0(0.00)	1(6.25)	
**BCLC stage**			0.748			0.324
A	8(17.39)	9(22.50)		0(0.00)	5(31.25)	
B	38(82.61)	31(77.50)		6(100.00)	11(68.75)	
**Child-Pugh grade**			1.000			1.000
A	41(89.13)	36(90.00)		5(83.33)	14(87.50)	
B	5(10.87)	4(10.00)		1(16.67)	2(12.50)	
**ALBI grade**			0.038			0.238
1	13(28.26)	21(52.50)		1(16.67)	9(56.25)	
2&3	33(71.74)	19(47.50)		5(83.33)	7(43.75)	
**AFP (IU/mL)**			0.978			0.601
≤400	31(67.39)	28(70.00)		5(83.33)	16(100.00)	
>400	15(32.61)	12(30.00)		1(16.67)	0(0.00)	
**PT extension time score**			0.315			1.000
1	39(84.78)	36(90.00)		6(100.00)	15(93.75)	
2	7(15.22)	3(7.50)		0(0.00)	1(6.25)	
3	0(0.00)	1(2.50)		0(0.00)	0(0.00)	
**Lobar involvement**			0.075			0.102
Unilobar	20(43.48)	26(65.00)		2(33.33)	13(81.25)	
Bilobar	26(56.52)	14(35.00)		4(66.67)	3(18.75)	
**Microsphere size (μm)**			0.686			0.560
70–150	6(13.04)	3(7.50)		0(0.00)	2(12.50)	
100–300	35(76.09)	33(82.50)		4(66.67)	11(68.75)	
300–500	5(10.87)	4(10.00)		2(33.33)	3(18.75)	
**Vascular lake**			0.089			1.000
Yes	5(10.87)	11(27.50)		1(16.67)	2(12.50)	
No	41(89.13)	29(72.50)		5(83.33)	14(87.50)	
**Capsule**			0.031			0.752
Yes	23(50.00)	30(75.00)		3(50.00)	11(68.75)	
No	23(50.00)	10(25.00)		3(50.00)	5(31.25)	
**Vascularization patterns**			0.171			0.222
type 1&2	9(19.57)	14(35.00)		0(0.00)	6(37.50)	
type 3&4	37(80.43)	26(65.00)		6(100.00)	10(62.50)	
**SACE grade**			0.375			0.071
1	2(4.35)	0(0.00)		1(16.67)	1(6.25)	
2	5(10.87)	5(12.50)		2(33.33)	0(0.00)	
3	38(82.61)	32(80.00)		3(50.00)	14(87.50)	
4	1(2.17)	3(7.50)		0(0.00)	1(6.25)	
**6 and 12 score**			0.272			0.017
>12	16(34.78)	8(20.00)		3(50.00)	1(6.25)	
>6 but ≤12	17(36.96)	16(40.00)		3(50.00)	6(37.50)	
≤6	13(28.26)	16(40.00)		0(0.00)	9(56.25)	

OR, objective response; PLT, blood platelet; BCLC, Barcelona Clinic Liver Cancer; ALBI, albumin-bilirubin; AFP, α-fetoprotein; SACE, Subjective Angiographic Chemoembolization Endpoint.

**FIGURE 3:**
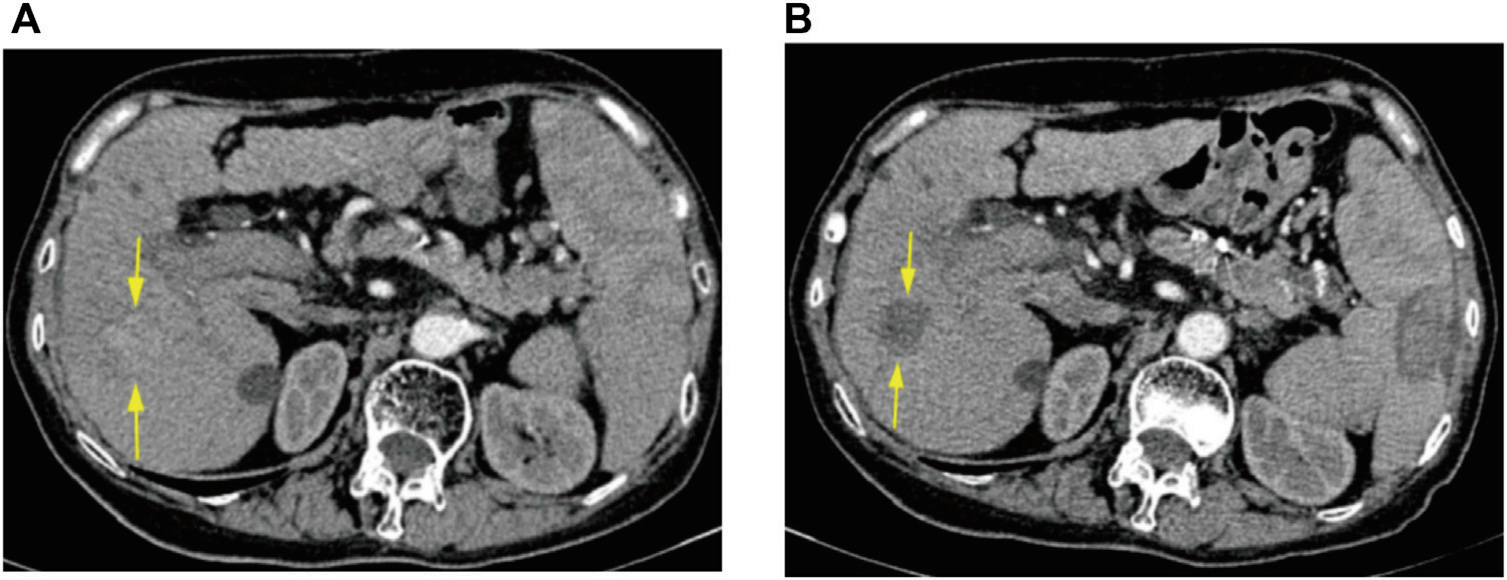
CT arterial phase images of a 75-year-old female patient before DEB-TACE and at 6 weeks postoperative re-examination. **(A)** Preoperative enhanced CT representing HCC in the S5 segment of the liver. **(B)** Postoperative enhanced CT demonstrating no enhancement of surviving tumor in the area of the original lesion, with efficacy assessed as CR.

**FIGURE 4:**
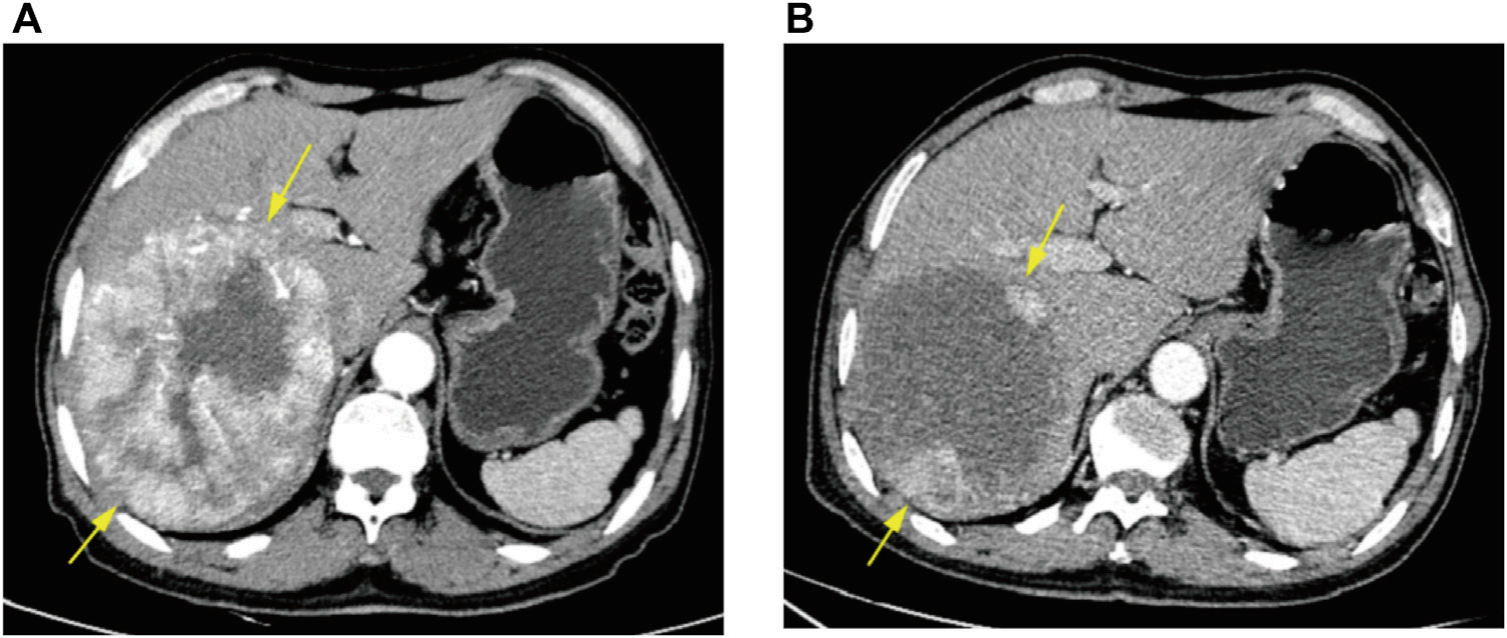
CT arterial phase images of a 66-year-old male patient before DEB-TACE and at 6 weeks postoperative re-examination. **(A)** Preoperative enhanced CT demonstrating massive HCC in the right lobe of liver. **(B)** Postoperative enhanced CT signifying nodular enhancement of the surviving tumor surrounding the wall of the original lesion area, with efficacy assessed as PR.

The response to initial DEB-TACE treatment in 108 HCC patients was analyzed based on the mRECIST criteria. Fifty-six patients (51.9%) in the original group who achieved initial OR (Figures 3, 4) and 52 (48.1%) who did not achieve OR were randomized into a training group (*n* = 86) and a validation group (*n* = 22) in an 8:2 ratio. In the training group, only the differences in the capsule and ALBI grade were significant (*p* < 0.05) and included in constructing the clinical-radiological model. The differences in serum albumin, maximum tumor diameter, and six-and-twelve scores in the validation group were statistically significant. Cohen’s Kappa test indicated good reliability of lobar involvement, vascular lakes, capsule, vascularization patterns, SACE, and initial treatment response, with Kappa values of 0.94, 0.87, 0.84, 0.91, 0.83, and 0.87, respectively. None of the 108 patients died within 1 month of treatment, and 81 of them (75%) presented with post-embolization syndrome to varying degrees. In accordance with the recently suggested adverse event categorization provided by the Standards of Practice Committee of the Society of Interventional Radiology (Khalilzadeh et al., 2017), the incidence of grade 1–2 post-embolization syndrome was 80.2% (65/81), and that of grade 3–4 post-embolization syndrome was 19.8% (16/81).

### 3.2 Construction of the radiomics model

**FIGURE 5:**
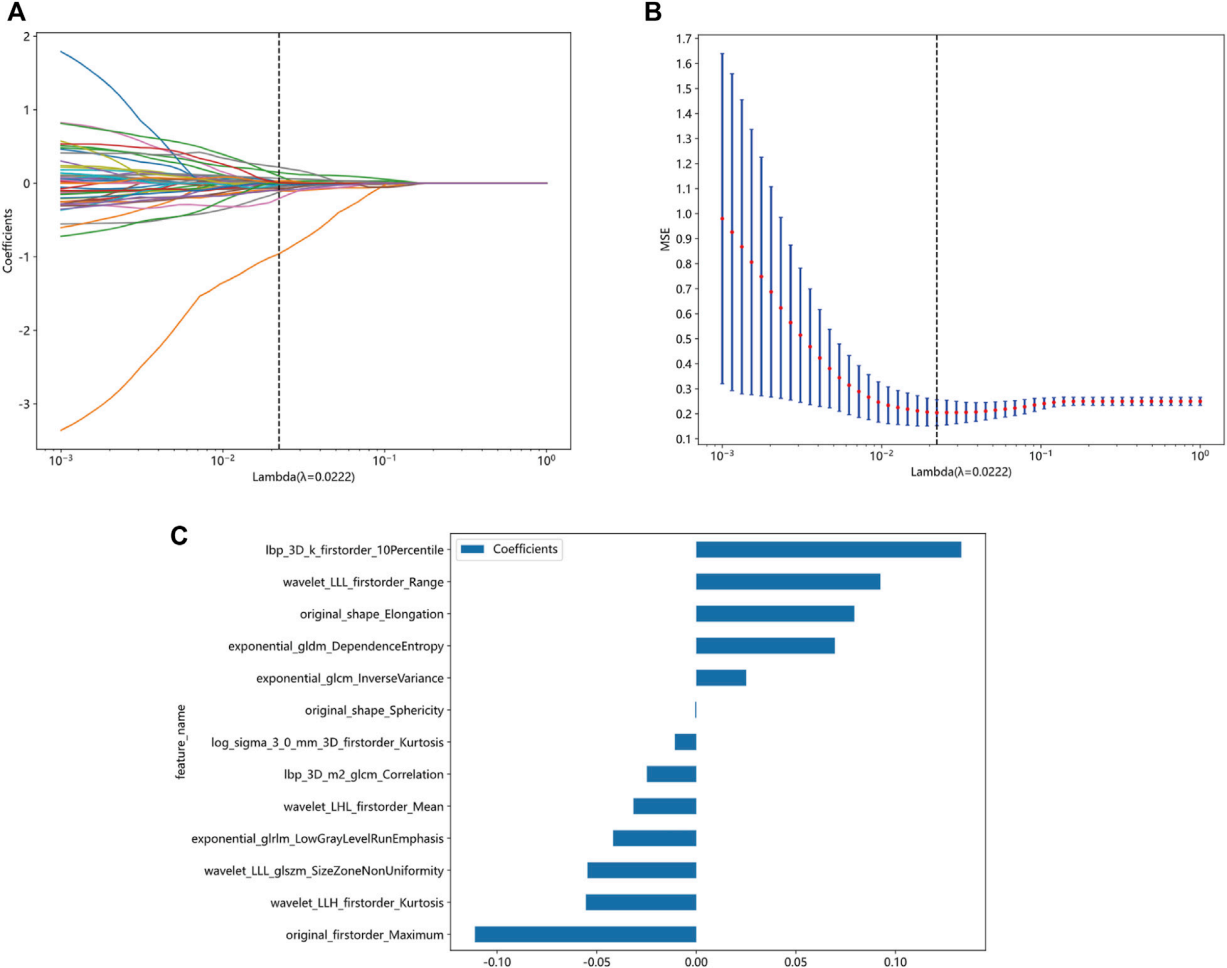
Screening of radiomics features based on the LASSO algorithm and histogram of radiomics scores based on selected features. **(A)** 10-fold cross-validation coefficient. **(B)** Mean square error of 10-fold cross-validation. **(C)** Histogram of radiomics scores based on selected features. LASSO, least absolute shrinkage and selection operator.

**FIGURE 6:**
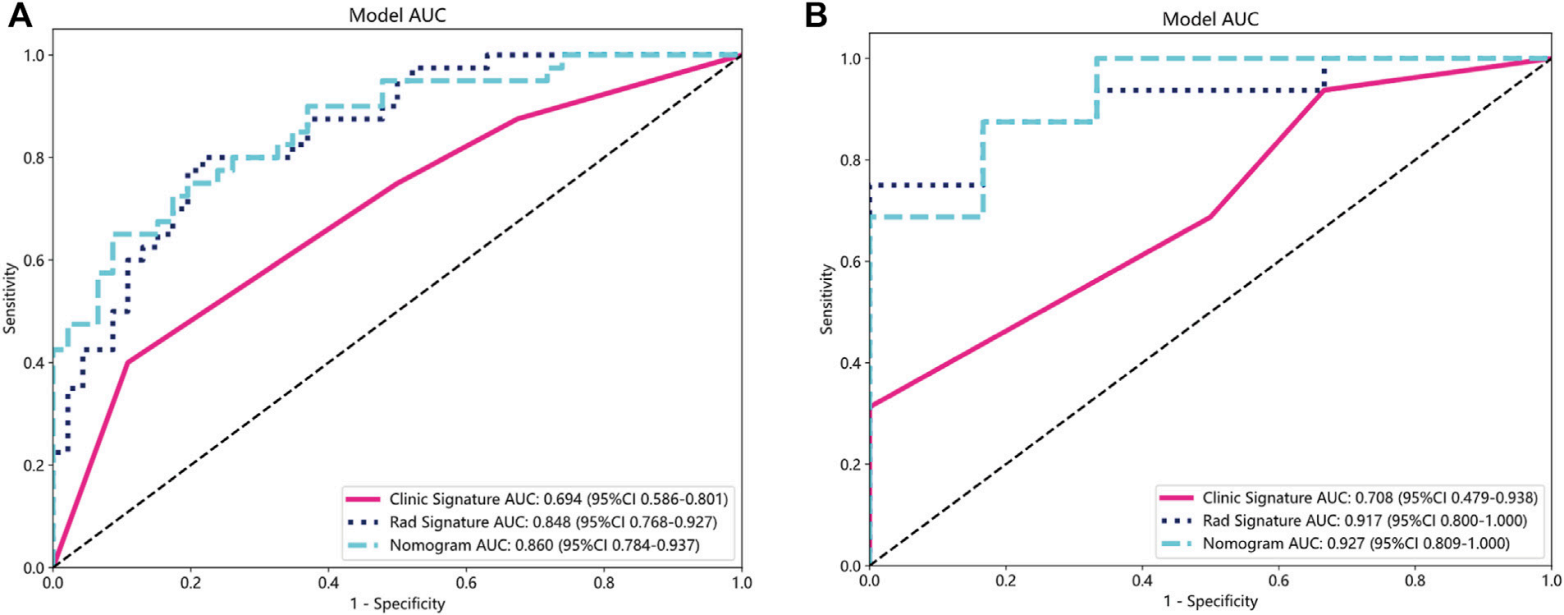
ROC curves for the clinical-radiological, radiomics, and integrated models in training **(A)** and validation groups **(B)**. ROC, receiver operating characteristic; AUC, the area under curve.

A total of 1834 radiomics features were extracted, comprising 360 first-order features, 14 shape-based features, and 1460 textural features. The textural features were categorized into five primary groups, namely, the gray-level size zone matrix (GLSZM), gray-level co-occurrence matrix (GLCM), gray-level run length matrix (GLRLM), neighborhood gray-tone difference matrix (NGTDM), and gray-level dependence matrix (GLDM). Following a series of screening procedures, a set of 13 features exhibiting non-zero coefficient values were ultimately retained. The LR model was employed in the LASSO technique to generate radiomics scores. These scores were derived from the coefficient values of the chosen features within the training group. Figure 5 presents the feature coefficients, the mean standard error (MSE) derived from the 10-fold cross-validation, and the coefficient values associated with the ultimately chosen non-zero features. The additional characteristics that underwent screening were employed in the development of a radiomics model, which was founded on the LR model. To prevent overfitting, a 5-fold cross-validation was employed to select the optimal parameters of the model, and to obtain final radiomics signatures. The radiomics model demonstrated that in the training group, the AUC was 0.848 (95% confidence interval (CI): 0.768–0.927). The investigation yielded sensitivity and specificity values of 0.800 and 0.783, respectively. Furthermore, the investigation determined the positive predictive value (PPV) to be 0.762 and the negative predictive value (NPV) to be 0.818. The AUC in the validation group was found to be 0.917, with a 95% confidence interval ranging from 0.800 to 1.000. The estimated values for sensitivity and specificity are 0.750 and 1.000, respectively. Furthermore, the PPV was determined to be 1.000, while the NPV was calculated to be 0.600. The findings are displayed in Table 2; Figure 6. Among the 13 features retained, the feature lbp_3D_k_firstorder_10Percentile contributed the most to predict the responsiveness of HCC patients to initial DEB-TACE treatment. Following is the calculation of the Rad score: Rad_score = 0.565103194542222 + (0.025064 × exponential_glcm_InverseVariance) + (0.069557 × exponential_gldm_DependenceEntropy) − (0.041746 × exponential_glrlm_LowGrayLevelRunEmphasis) + (0.133036 × lbp_3D_k_firstorder_10Percentile) − (0.024827 × lbp_3D_m2_glcm_Correlation) − (0.010773 × log_sigma_3_0_mm_3D_firstorder_Kurtosis) − (0.111148 × original_firstorder_Maximum) + (0.079339 × original_shape_Elongation) − (0.000535 × original_shape_Sphericity) − (0.031579 × wavelet_LHL_firstorder_Mean) − (0.055422 × wavelet_LLH_firstorder_Kurtosis)+ (0.092430 × wavelet_LLL_firstorder_Range) − (0.054587 × wavelet_LLL_glszm_SizeZoneNonUniformity).

**TABLE 2:** Prediction performance of the three models in the training and validation groups.

Model	Training cohort (*n* = 86) AUC(95%CI)	Sensitivity	Specificity	PPV	NPV	Test cohort (*n* = 22) AUC(95%CI)	Sensitivity	Specificity	PPV	NPV
Clinical-radiological model	0.694(0.586–0.801)	0.400	1.000	0.762	0.631	0.708(0.479–0.938)	0.312	1.000	1.000	0.353
Radiomics model	0.848(0.768–0.927)	0.800	0.783	0.762	0.818	0.917(0.800–1.000)	0.750	1.000	1.000	0.600
Combined model	0.860(0.784–0.937)	0.650	0.913	0.867	0.750	0.927(0.809–1.000)	0.875	0.833	0.933	0.714

AUC, area under curve; CI, confidence interval; PPV, positive predictive value; NPV, negative predictive value.

### 3.3 Clinical-radiological and integrated model construction

Clinical-radiological models were constructed for clinical or radiological features with *p* < 0.05 in the training group. Following analysis of variance, only the baseline capsule and ALBI grade met the conditions and were used for the construction of the clinical-radiological signature. We constructed the model LR in machine learning, similar to the aforementioned approach. In the training group, the clinical-radiological model demonstrated an AUC of 0.694, accompanied by a 95% CI: 0.586–0.801. The model demonstrated a sensitivity value of 0.400 and a specificity value of 1.000. Furthermore, the PPV and NPV were calculated to be 0.762 and 0.631, correspondingly. The AUC in the validation group was determined to be 0.708, accompanied by a 95% CI: 0.479–0.938. The diagnostic test exhibited a sensitivity of 0.312 and a specificity of 1.000. Furthermore, the PPV and NPV were determined to be 1.000 and 0.353, correspondingly, as presented in Table 2; Figure 6.

**FIGURE 7:**
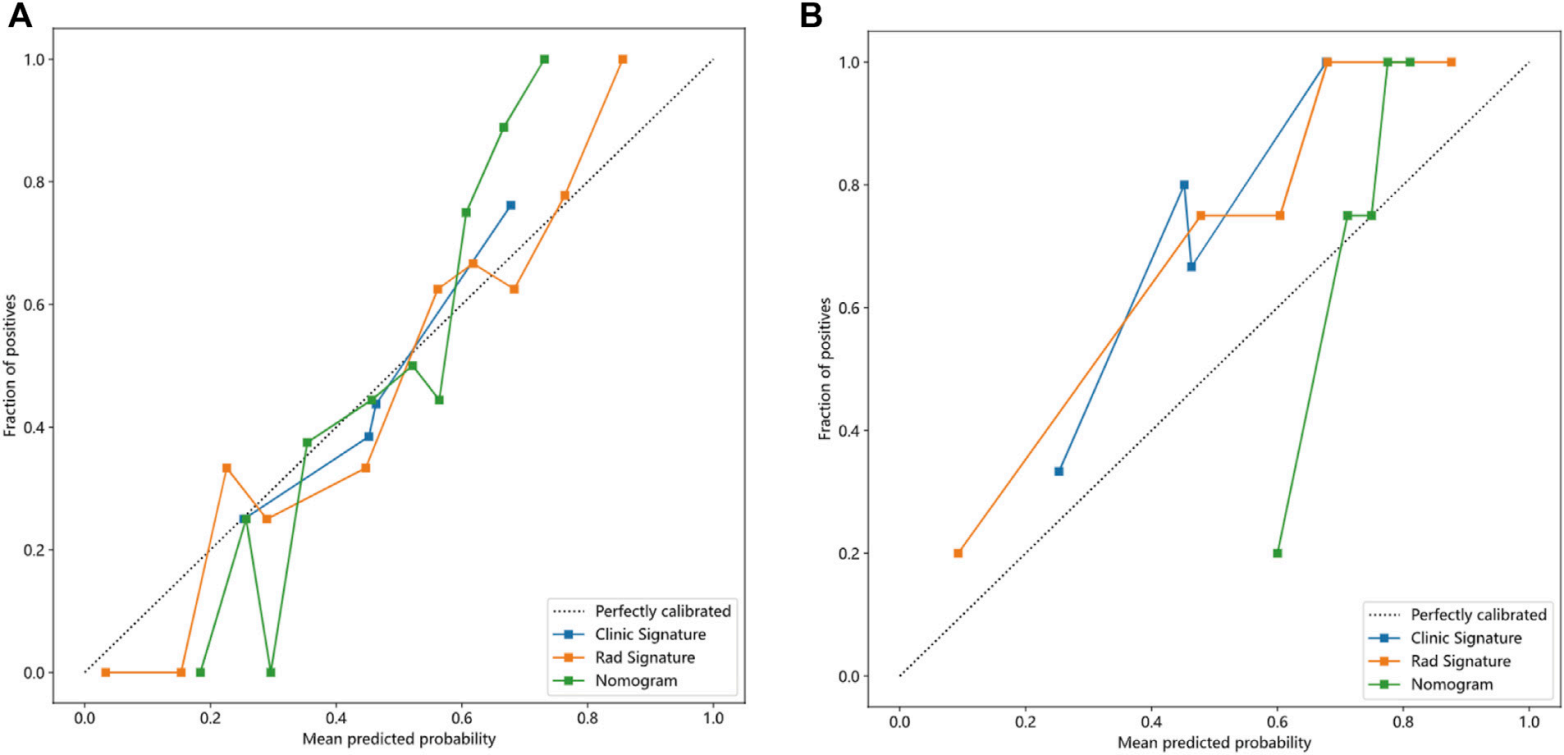
Calibration curves of the clinical-radiological, radiomics, and integrated models in training **(A)** and validation groups **(B)**.

**FIGURE 8:**
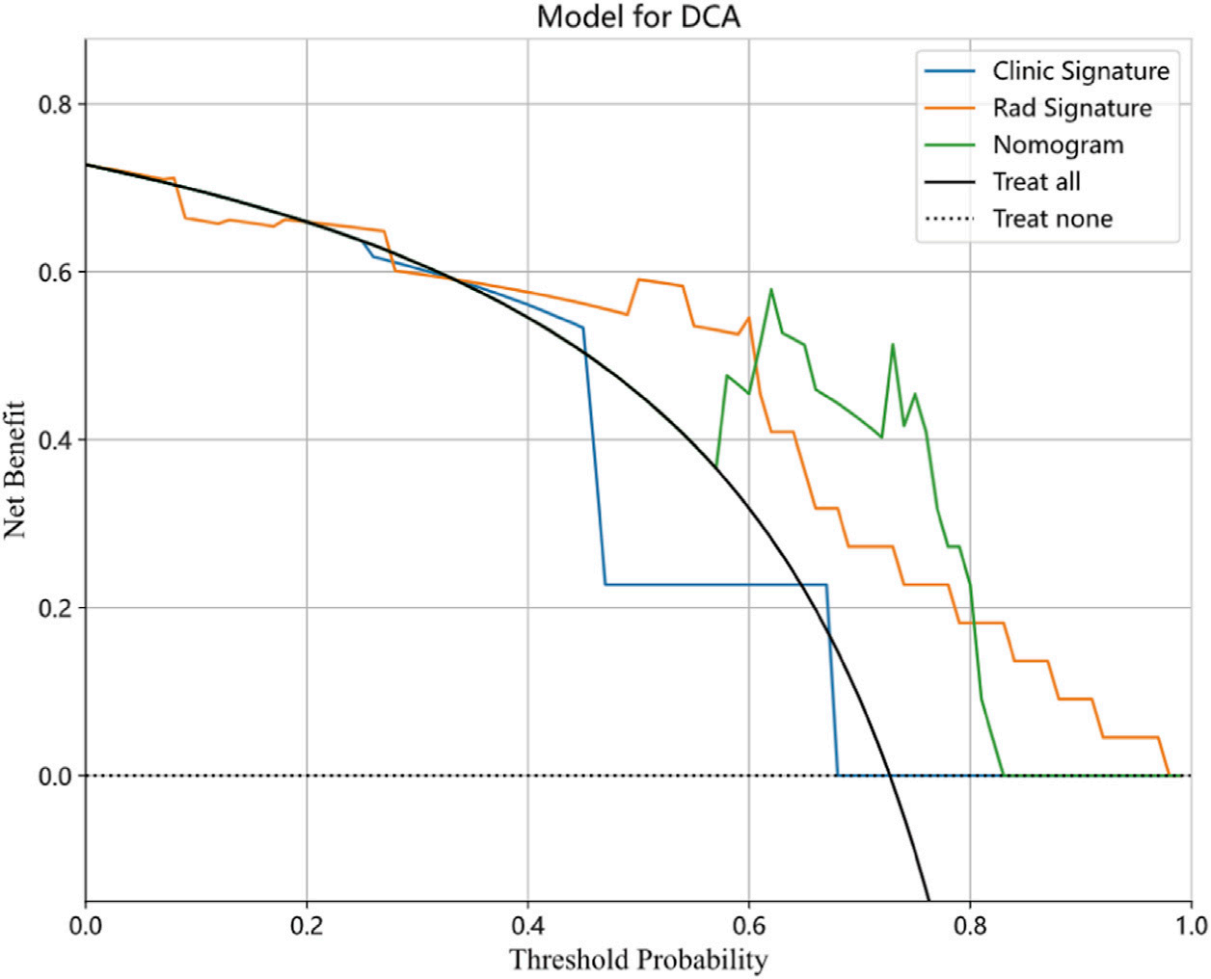
Decision curve analysis for the clinical-radiological, radiomics, and integrated model in the validation cohort.

**FIGURE 9:**
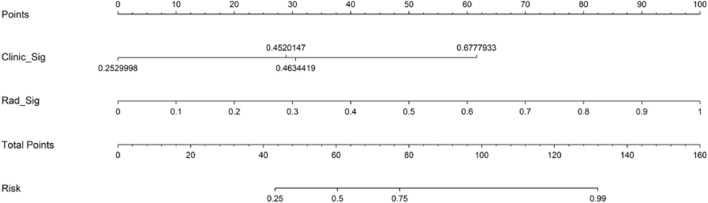
Nomogram for predicting objective response to initial DEB-TACE in HCC individuals. DEB-TACE, drug-eluting bead transcatheter arterial chemoembolization; HCC, hepatocellular carcinoma.

The nomogram derived from the LR algorithm by integrating radiomics and clinical-radiological features (Figure 9) exhibited superior performance. In the group used for training, the AUC was found to be 0.860, 95% CI: 0.784–0.937. The sensitivity of the model was determined to be 0.650, while the specificity was measured to be 0.913. Furthermore, the PPV and NPV were calculated to be 0.867 and 0.750, respectively. The AUC in the validation group was determined to be 0.927 (95% CI: 0.809–1.000). The sensitivity and specificity values were 0.875 and 0.833, respectively. Moreover, the PPV and NPV were calculated to be 0.933 and 0.714, respectively. These results can be observed in Table 2; Figure 6. The DeLong test was employed to conduct a comparison of the AUC between the different models. In the validation group, significant alterations in the AUC values were observed between the clinical-radiological and the integrated models (*p* = 0.042). However, no significant distinction was detected between the clinical-radiological model and the radiomics model (*p* = 0.079), and no significant variation was noted between the radiomics and the integrated models (*p* = 0.734). The nomogram’s calibration curves demonstrated a significant higher degree of agreement between the anticipated response to the initial DEB-TACE treatment and the actual response observed in the training and validation groups. The *p*-values obtained from the Hosmer-Lemeshow test for the clinical-radiological model, radiomics model, and integrated model were 0.114, 0.186, and 0.128, respectively. These results suggest that the nomogram exhibited enhanced concordance in the training and validation groups. Figure 7 illustrates the calibration curves for each model within the training and validation groups. The findings from the DCA reveal that the integrated model exhibited a positive net benefit when considering the threshold probability range of 58%–83%. Furthermore, the integrated model demonstrated a greater net benefit in comparison to the radiomics model within the threshold probability range of 61%–81% (see Figure 8). However, the radiomics model had a wider range of threshold probabilities with good net benefit, at threshold probabilities of 20%–28% and 34%–98% (Figure 8). Figure 9 shows a nomogram integrating the radiomics and clinical-radiographic features, with the total score reflecting the likelihood of achieving OR following initial DEB-TACE in HCC patients.

## 4 Discussion

The integration of targeted therapy and immunotherapy has demonstrated significant efficacy in HCC treatment, TACE is still endorsed as the standard treatment for intermediate-stage HCC (Heimbach et al., 2018; Vogel et al., 2021; Reig et al., 2022). DEB-TACE allows for higher intratumoral chemotherapeutic agent concentrations, longer retention times, lower systemic plasma chemotherapeutic agent levels, and reduced hepatotoxicity compared to cTACE, which lacks standardization (Lewis et al., 2006a; Lewis et al., 2006b; Hong et al., 2006; Poon et al., 2007; Varela et al., 2007). Thus, an increasing share of DEB-TACE use has been observed in clinical practice.

Several investigations have concluded that overall survival (OS) should be the gold standard for assessing the efficacy of TACE for HCC (European Association for the Study of the Liver (EASL), 2018; Heimbach et al., 2018; Vogel et al., 2019). However, collecting OS data requires a huge number of samples and a long period of follow-up and may be affected by sequential therapies, making clinical research difficult if OS is the only study endpoint (Llovet et al., 2019; Llovet et al., 2021). OR for local treatment can be determined at early stages and is strongly correlated with OS (Kudo, 2018; Han et al., 2020). Consequently, some researchers have suggested that mRECIST-based OR could be a reliable surrogate endpoint for OS (Gillmore et al., 2011; Memon et al., 2011; Prajapati et al., 2013; Llovet and Lencioni, 2020). However, the ORRs reported in current studies have been inconsistent, with variation as much as 30% (Llovet and Lencioni, 2020); this may be attributed to inconsistencies across studies in the time points at which response was assessed, especially in clinical practice where routinely repeated “on-demand” TACE therapy is often required. The initial OR and the best OR are available in the analysis of clinical studies, and it remains controversial which OR is more reasonable as a surrogate endpoint (Wang et al., 2015). However, initial OR is immediate, and its role in predicting prognosis and clinical decision-making cannot be underestimated (Xia et al., 2022). In addition, intermediate-stage HCC patients are highly heterogeneous, and the clinical benefits of DEB-TACE treatment may vary widely among them (Kudo et al., 2020). One study reported no variation in survival between TACE non-responders and untreated patients, and in cases where OR was not achieved with initial TACE, repeated TACE is not recommended (Llovet et al., 2002). Therefore, it is important to effectively screen patients with HCC prior to initial DEB-TACE treatment. Patients predicted preoperatively to have a higher probability of OR should be treated with DEB-TACE, whereas other HCC patients should be treated systematically in accordance with the treatment stage migration strategy.

Radiomics converts a large number of image features into high-dimensional data that enables objective and precise analysis of the CT value of each pixel within a lesion and detection of subtle variations in density within a lesion to help physicians make medical decisions (Lubner et al., 2017). Although studies have been conducted on deep learning, radiomics, and integrated models for predicting survival and prognosis or efficacy in intermediate or advanced HCC patients who received TACE (Kim et al., 2018; Kong et al., 2021; Zhao et al., 2021; Wang et al., 2022; Sun et al., 2023), few studies have explored predictive models related to DEB-TACE. Therefore, the present investigation focused on the initial OR of DEB-TACE for the prediction of efficacy.

The initial ORR of 51.9% in the present study was lower than 64.5% in a previous TACE-related study by Xia et al. (Xia et al., 2022) and similar to the 50% ORR reported in research study by Georgiades et al. (Georgiades et al., 2012). However, both these studies involved cTACE treatment. In the clinical-radiological model of the present study, the capsule and ALBI grade were predictors of initial OR in DEB-TACE. The loss of tumor capsules has been closely linked to microvascular invasion in HCC, which could be a sign of more aggressive tumors and poorer survival rates (Zheng et al., 2018; Kim et al., 2019; Ji et al., 2020). It has been proposed that the existence of a capsule in HCC may be linked to an increased incidence of necrosis (Odisio et al., 2014). In addition, the ALBI scoring system is considered a straightforward and unbiased model utilized to evaluate liver function in HCC patients (Johnson et al., 2015). HCC patients who exhibit elevated ALBI grades are more likely to experience impaired liver function, lower tumor biological behavior grades, and a deteriorated systemic status and prognosis. Our results are also consistent with these findings.

CT is an imaging modality that does not require invasive procedures and has enjoyed widespread clinical use for tumor diagnosis, treatment plan selection, and efficacy monitoring (Chen et al., 2021). Compared to conventional CT features, radiomics features allow for more objective and quantitative information on intratumoral heterogeneity at a low cost (Lambin et al., 2012). The data correlate with underlying gene expression patterns and are strongly related to tumor invasiveness at the cellular level (Lambin et al., 2012). Radiomics enables the prediction of clinical endpoints, including survival and treatment response, and can be integrated with clinical data and genetic information to construct and validate various models through machine learning or artificial intelligence for clinical application (Kumar et al., 2012; Lambin et al., 2012; Aerts et al., 2014; Choi et al., 2016; Lubner et al., 2017; Sala et al., 2017). In this investigation, we developed and verified several models for forecasting the treatment response to initial DEB-TACE for HCC. Specifically, we constructed a clinical-radiological, a radiomics, and an integrated models that incorporated both clinical, radiological, and CT radiomics features. The 13 radiomics features that were screened to reflect the pattern or spatial distribution of voxel intensity within the tumor can be used as parameters to capture tumor heterogeneity. The three most influential features that contributed to the outcome were “lbp_3D_k_firstorder_10Percentile,” “original_firstorder_Maximum,” and “wavelet_LLL_firstorder_Range.” The first three most influential features are all first-order statistics, mainly describing the distribution of voxel intensities in the lesion. The higher the median value, the denser the distribution of voxel intensities in the lesion, indicating a more dense lesion. The AUC values of the integrated model in both the training and validation groups were 0.860 (95% CI: 0.784–0.937) and 0.927 (95% CI: 0.809–1.000), respectively. A significant discrepancy was noted between the integrated model and the clinical-radiological model (*p* = 0.042) within the validation group. Nonetheless, no significant distinction existed between the integrated model and the radiomics model (*p* = 0.734) within the identical group. The predictive performance of the integrated model, which integrated clinical, radiological, and radiomics features, was superior to that of models utilizing only individual data types in both the training and validation groups. Furthermore, the integrated model accurately predicted the response to initial DEB-TACE treatment. The findings of our study align with those reported by Zhao et al. (2021), who showed that, in the training group, a model that integrates three-stage enhanced MRI radiomics scores with clinical-radiological risk factors (total bilirubin, tumor morphology, and tumor capsule) demonstrated significantly higher AUC values than a clinical-radiological model in predicting objective outcomes after TACE (0.878 vs. 0.744, *p* = 0.003). Nevertheless, no statistically significant variation existed in the AUC between the two models in the validation group (*p* = 0.239) (Zhao et al., 2021). Moreover, the researchers discovered that there was no statistically significant disparity in the AUC between the integrated and the radiomics models (*p* = 0.155, 1.000) in both the training and validation cohorts of their investigation. Likewise, no substantial disparity in the AUC was observed between the clinical-radiological and the radiomics models (*p* = 0.148, 0.344) (Zhao et al., 2021), and this finding is similar to our results. Another model integrating MRI radiomics and clinical features had a greater ROC than an MRI radiomics-only model for predicting local treatment outcomes in patients with liver cancer, but the variation was not significant (0.867 vs. 0.833, respectively, *p* = 0.573) (Wang et al., 2023). The findings of the current investigation suggest that the radiomics model exhibited superior predictive capabilities compared to the clinical-radiological model, albeit without a statistically significant distinction between the two (AUC: 0.917 vs. 0.708, respectively; *p* = 0.079); it may also suggest a potential application of radiomics in the prediction of treatment response to initial DEB-TACE for HCC. A study on prognosis prediction following hepatic arterial infusion chemotherapy (HAIC) suggests that radiomics may be more valuable than clinical indicators for predicting prognosis after HAIC for unresectable HCC (Zhao et al., 2022). We also found that The AUC of the validation group in our research model is higher than the training group. We believe that possible reasons for this result include: high model complexity, small dataset size, potential use of features in the training process that were not present in the validation group, greater impact of certain features in the validation group on model performance, and differences in data distribution between the training and validation groups. Although the data in the validation group may have contributed to this result when calculated based on the converged model, we believe that the main reason is likely the small size of the dataset in our research. Therefore, further multicenter studies are needed in the future to increase the sample size. Additionally, we created a nomogram depending on the integrated model, which can be utilized in clinical practice. By adding the scores corresponding to clinic and radiomic signatures, the corresponding risk value of the total score can be used as the risk prediction value for ORR in HCC patients after initial DEB-TACE treatment. DCA curves indicated that the integrated model demonstrated good net benefit in the threshold probability range of 58%–83%. This indicates that within the aforementioned threshold range, the decision curve of the integrated model was positioned above both the “none” and “all” lines. This suggests that if the model were employed for clinical decision-making at this juncture, it could yield a higher net benefit in the population compared to the “all ORR” or “none ORR” predictive approaches. This serves as evidence that the model possesses a greater practical clinical application value. Therefore, this means that based on our nomogram, it can help clinical doctors to select HCC patients who are most suitable for DEB-TACE treatment, and promote the early implementation of alternative treatments for patients who are not ideal candidates. This study represents the inaugural attempt to develop a nomogram by integrating clinical, radiological, and CT radiomics characteristics to predict the treatment response of initial DEB-TACE for HCC; the results demonstrate good discrimination, consistency, and clinical utility. In addition, some scholars had used CT radiomics signatures based on lung cancer datasets to predict head and neck squamous cell carcinoma and renal cell carcinoma, and believe that radiomics signatures based on CT may be able to predict overall survival rates for different cancer sites (Le et al., 2023). In furthermore, scholars have developed a multiscale modelling framework to explain the microstructurally driven heterogeneity of permeability and porosity in brain tissue, aiming to better understand the importance of drug transport in the brain and the response of brain tissue to infusion pressure, and to predict the flow path and concentration distribution of drugs (Yuan et al., 2022). In the future, we can further explore the extraction of microstructural features of liver tissue and tumors from patients based on CT radiomics, to validate whether the radiomics signatures of HCC can be used to predict tumors in different organs. Alternatively, we can attempt to study the potential relationship between drug infusion range and kinetics and these microstructural features through multiscale model. This can guide the clinical selection of appropriate microsphere size, catheter type, drug delivery rate, and pressure conditions to improve the clinical benefits obtained by patients from DEB-TACE treatment.

The present study has the following limitations. First, the limited sample size and potential selection bias restrict the practical performance of the model, where the small data size may be the fundamental reason for the model’s validation group AUC being higher than the training group AUC. Second, this retrospective, single-center study was not externally validated. Third, target area segmentation was performed manually, which is time-consuming and inevitably involves human error. Last, no application of more sophisticated techniques such as deep learning. Therefore, an automatic and reliable segmentation method is needed for future clinical practice, as well as validation of the performance of the proposed prediction model in a large, multi-center prospective study. Also further research such as deep learning is needed in the future.

## 5 Conclusion

The integrated model could better predict the treatment response of initial DEB-TACE for HCC. It may help clinicians select patients with HCC that are ideally suited for DEB-TACE treatment, facilitate early implementation of alternative treatments for non-ideal patients, and support the formulation of individualized treatment plans for patients.

## Figures and Tables

**Table:** 

**HCC**	hepatocellular carcinoma
**TACE**	transcatheter arterial chemoembolization
**DEB-TACE**	drug-eluting beads transcatheter arterial chemoembolization
**mRECIST**	modified Response Evaluation Criteria in Solid Tumors
**LR**	logistic regression
**ROC**	receiver operating characteristic
**DCA**	decision curve analysis
**AUC**	area under curve
**PPV**	positive predictive value
**NPV**	negative predictive value
**ESMO**	European Society for Medical Oncology
**AASLD**	American Association for the Study of Liver Diseases
**BCLC**	Barcelona Clinic Liver Cancer
**CSCO**	Chinese Society of Clinical Oncology
**ECOG**	Eastern Cooperative Oncology Group
**SACE**	Subjective Angiographic Chemoembolization Endpoint
**ALBI**	albumin-bilirubin
**CR**	complete response
**PR**	partial response
**SD**	stable disease
**PD**	progressive disease
**OR**	objective response
**ORR**	objective response rate
**VOI**	volume of interest
**ICCs**	intraclass correlation coefficients
**GLCM**	gray-level co-occurrence matrix
**GLDM**	gray-level dependence matrix
**GLRLM**	gray-level run length matrix
**GLSZM**	gray-level size zone matrix
**NGTDM**	neighborhood gray-tone difference matrix
**LASSO**	least absolute shrinkage and selection operator
**MSE**	mean square error
**PLT**	blood platelet
**AFP**	α-fetoprotein
**CI**	confidence interval
**OS**	overall survival
**HAIC**	hepatic arterial infusion chemotherapy

## Data Availability

The original contributions presented in the study are included in the article/Supplementary Material, further inquiries can be directed to the corresponding author.
